# Physiological responses to full and segmented duet routines in elite artistic swimmers

**DOI:** 10.1371/journal.pone.0333791

**Published:** 2025-10-07

**Authors:** Xavier Iglesias, Lara Rodríguez-Zamora, Marta Carrasco-Marginet, Alfredo Irurtia, Ferran A. Rodríguez, Ignacio Fernández-Jarillo, Diego Chaverri

**Affiliations:** 1 Grup de Recerca en Ciències de l’Esport INEFC Barcelona (GRCEIB), Institut Nacional d’Educació Física de Catalunya (INEFC), Universitat de Barcelona (UB), Barcelona, Spain; 2 School of Health and Medical Sciences, Division of Sport Sciences, Örebro University, Örebro, Sweden; Mae Fah Luang University School of Anti Aging and Regenerative Medicine, THAILAND

## Abstract

Artistic swimming combines prolonged breath-hold periods with high-intensity movements, resulting in unique physiological demands. Direct measurement of key variables such as oxygen uptake (VO₂) during routines is limited by frequent immersion. However, VO₂ monitoring is essential for understanding the balance between aerobic and anaerobic energy contributions, guiding training strategies and reducing injury risk. This study aimed to analyze the acute physiological responses, VO₂, blood lactate concentration, and heart rate, during free duet routines in elite artistic swimmers, using a segmented protocol that emphasized the two longest apneas. Sixteen elite artistic swimmers performed both complete and segmented versions of the routine. VO₂ was estimated using retro-extrapolation, while lactate was measured after each phase, and heart rate was continuously monitored. The protocol included six measurement points: pre-routine, pre- and post-apnea 1 and 2, and post-routine. VO₂ increased rapidly, reaching nearly 90% of VO₂ peak within 67 seconds (mean: 61.8 ± 15.1 mL·min ⁻ ¹·kg ⁻ ¹). Blood lactate concentration rose progressively, peaking at 5.93 ± 1.41 mmol·L ⁻ ¹. Heart rate exhibited large fluctuations, with a maximum of 203.8 ± 5.0 beats·min ⁻ ¹ and a minimum of 71.9 ± 16.6 beats·min ⁻ ¹, reflecting a bradycardic response during apneas. No significant changes were observed in VO₂ or lactate between pre- and post-apnea values, as measured around the two longest apneas within the routine. These findings suggest that, under the specific conditions of this study, short-duration apneas (< 20 s) may be insufficient on their own to elicit distinct physiological shifts. However, the progressive increases observed in blood lactate and heart rate throughout the full routine suggest that the overall physiological load may be influenced more by sustained exercise intensity and the cumulative effect of repeated apneas than by isolated breath-hold events.

## Introduction

Artistic swimming (AS) is an aesthetic sport that combines high physical demand with choreography, technical precision and visual presentation [1]. Competitive events include Women’s and Men’s Solo, Duet, Mixed Duet, Open Team, Open Free Combination, and Open Acrobatic routines. At the Olympic Games, however, events are limited to the Women’s Duet (Technical and Free), and the Open Team (Technical, Free, and Acrobatic) [2].

The uniqueness of artistic swimming lies in the combination of intense physical exertion with complex technical elements performed under intermittent and often prolonged breath-hold periods. These apneas place a substantial physiological load on the cardiovascular system and on glycolytic metabolism [3]. They trigger the so-called diving response, a physiological adaptation characterized by peripheral vasoconstriction, reduced heart rate, and increased blood pressure [4,5]. At the metabolic level, this response shifts energy production toward anaerobic glycolysis, leading to increased lactate accumulation [5–7].

Several physiological markers have been used to assess responses during AS, such as oxygen uptake (VO₂), heart rate (HR), heart rate variability, blood lactate concentration, and muscle oxygen saturation [3,6–9], though most studies have primarily focused on HR and lactate [3,7,8].

This physiological response is particularly complex due to the so-called ‘autonomic conflict’, characterized by the simultaneous activation of parasympathetic (vagal) and sympathetic pathways, which exert opposing effects on cardiac function. This dynamic is reflected in the extreme heart rate fluctuations observed during routines, where swimmers may reach near-maximal HR values during surface movements (191 ± 15 beats·min ⁻ ¹), followed by abrupt decreases during apnea phases (82 ± 27 beats·min ⁻ ¹) [3].

Breath-hold control represents a fundamental component of artistic swimming routines, accounting for up to 50% of total routine time [8,10,11]. During these periods, swimmers perform complex and physically demanding movements while holding their breath, eliciting a physiological response comparable to that observed in other apnea-based aquatic disciplines, such as freediving [6]. This includes a pronounced bradycardic response as an oxygen-conserving mechanism, along with peripheral vasoconstriction that redistributes blood flow toward vital organs such as the brain and heart [12]. Performing high-intensity figures under breath-hold conditions has been linked to increased lactate production [13]. Moreover, a correlation has been reported between the frequency and duration of apnea periods and blood lactate accumulation [3].

These breath-hold phases are associated with hypercapnia and metabolic acidification (↓pH, ↓ HCO₃ ⁻ , ↑ K⁺), as well as progressive increases in lactate when they coincide with intense underwater muscular contractions [1,3,8]. Such acute alterations can compromise cognitive function and decision-making [14,15], increasing the risk of synchronization and execution errors in duet performance, and, in extreme cases, even syncope [13,15,16].

Aerobic capacity (VO₂ max/VO₂ peak) is often considered a performance determinant in AS, yet evidence is mixed: some studies report positive associations with routine scores, especially in solo events, whereas others do not, likely reflecting the use of non-sport-specific protocols that fail to replicate intermittent apneas and acrobatic intensity [8,17–19]. Crucially, within-routine VO₂ at the end of apnea periods and its fluctuations relative to pre-apnea values within the same routine have not been documented. This unaddressed question, the characterization of VO₂ dynamics around breath-hold phases, represents a central gap for understanding the aerobic–anaerobic balance under intermittent load and its translation to duet performance [3,8,14].

Direct, continuous VO₂ measurement underwater during complex AS routines is technically infeasible. Accordingly, retro-extrapolation [20] is an established procedure in aquatic exercise physiology and has already been applied in AS and related contexts [1,8,21]. Prior work has estimated VO₂ peak during simulated routines, reporting values of approximately 40–50 mL·min^−1^·kg^−1^, comparable to incremental cycling tests and indicative of a substantial aerobic load under ecologically valid conditions [8]. Building on this established approach, the present study estimates VO₂ at critical moments flanking the longest apneas within a segmentation-based, in-routine protocol that time-locks estimates to discrete apnea phases to characterize VO₂ dynamics around breath-holds and to clarify their potential implications for duet synchronization and execution.

Other studies have assessed VO₂ peak in artistic swimmers using non-specific protocols, such as swimming, cycling, or treadmill tests [17–19,22,23], typically reporting values between 43 and 51 mL·min ⁻ ¹·kg ⁻ ¹. Notably, Yamamura et al. [18] used a front-crawl protocol and still observed positive associations with competition scores, underscoring that non–sport-specific tests can nevertheless capture aspects of aerobic fitness. Roby et al. [22], using a tethered swimming test, found limited differences in VO₂ max between artistic swimmers and untrained individuals, suggesting a limited contribution of aerobic capacity in that context. In contrast, Poole et al. [17] and Viana et al. [19] reported significant correlations between VO₂ max, often assessed using cycle ergometers, and solo routine performance. Additionally, Chatard et al. [23] linked VO₂ peak to technical routine scores and observed declines in aerobic capacity after five weeks of technical training alone. Taken together, these findings offer mixed evidence on the role of aerobic fitness in artistic swimming and point to the limitations of non-specific testing, highlighting the need for sport-specific, ecologically valid protocols that resolve the acute metabolic effects of breath-hold phases and their performance consequences in duet routines, with VO₂ estimates used to contextualize aerobic-system engagement.

Therefore, this study aimed to analyze the physiological responses, oxygen uptake, heart rate, and blood lactate concentration, during the execution of a free duet routine in elite artistic swimmers. To this end, the routine was experimentally segmented to isolate and evaluate the specific physiological impact of the two longest apnea periods within the choreography.

We hypothesized that (H1) heart rate, oxygen uptake, and blood lactate would increase as the routine progressed across phases, and (H2) these physiological indicators would differ between pre- and post-apnea measurements around the two longest apneas.

## Materials and methods

### Design

A controlled quasi-experimental design was implemented to assess physiological responses during complete and segmented execution of free duet routines in elite artistic swimmers. The methodology maintained ecological validity and incorporated a repeated-measures design, enabling the analysis of physiological responses during key phases of the routine, particularly those involving the two longest apneas.

### Participants

Sixteen elite artistic swimmers (eight duets), including five World Championship medalists, participated in the study. The swimmers’ characteristics are presented in Table 1. All participants had over 7 years of competitive experience and trained more than 30 hours per week. Data collection took place between May 22 and July 11, 2011. Informed consent was obtained from all participants prior to their inclusion in the study, with additional consent from legal guardians in cases where participants were under 18 years of age. The study was conducted in accordance with the principles of the Declaration of Helsinki and adhered to current ethical standards for research in sport and exercise science [24]. The protocol received ethical approval from the Clinical Research Ethics Committee of Sport in Catalonia (reference: 2607-LA).

**Table 1:** Descriptive characteristics of the participating artistic swimmers.

Free Duet (n = 16 artistic swimmers)			
Age (years)	16.5	±	2.5
Height (cm)	166.7	±	7.4
Body mass (kg)	53.0	±	7.6

Note. Values are presented as mean ± standard deviation (SD).

The sample size (n = 16) is consistent with previous studies, which have used similar or smaller samples for the analysis of physiological and performance-related variables in this discipline [4,7,8,18,19,21]. The limited availability of high-level athletes and the need for sample homogeneity justify the selected sample, enhancing the ecological validity of the study. Despite the small elite sample, post hoc analyses confirmed that statistical power was sufficient (≥ 91%) to detect medium-to-large effects across key physiological variables.

### Protocol

All testing sessions were conducted in three different swimming pools affiliated with the Royal Spanish Swimming Federation: CAR Sant Cugat, Joaquín Blume Residence (Esplugues de Llobregat, Barcelona) and M-86 Swimming Center (Madrid). Water temperature was consistent across all testing sites, ranging from 26 °C to 27 °C. Athletes were evaluated in their usual duet pairings, following the same routine structure used in competition. Testing was conducted during the preparatory phase for the Olympic Games and the Junior World Championships, ensuring the ecological validity of performance conditions. All routines were memorized and executed by both swimmers at their habitual competition pace, with timing and intensity inherently guided by the accompanying music. The music was played through the speaker system. Additionally, the national team coach supervised all performances to ensure that each execution met the required technical quality and intensity standards.

**Fig 1:**
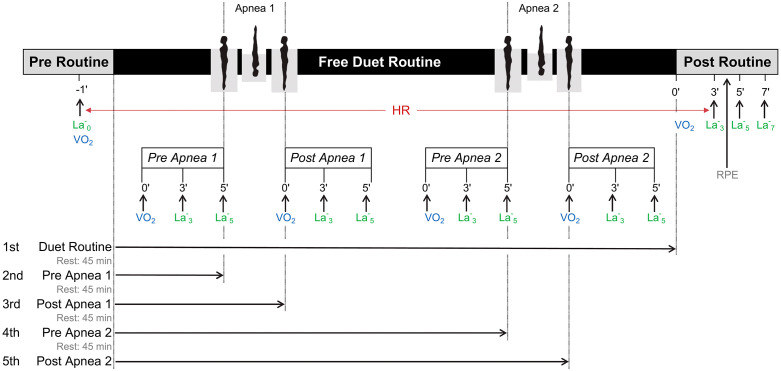
Schematic representation of the experimental protocol used to assess physiological responses (VO₂, blood lactate, and HR) during complete and segmented phases of a free duet artistic swimming routine. *Note.* The timeline is divided into Pre-Routine, Free Duet Routine, and Post Routine phases. Two apnea phases (Apnea 1 and Apnea 2) are highlighted within the routine. Heart rate (HR) was continuously monitored throughout. Measurements for oxygen uptake (VO₂) and blood lactate concentration (La₃, La_5,_ La_7_) were collected at multiple time points following key moments: Pre Apnea 1, Post Apnea 1, Pre Apnea 2, Post Apnea 2, and Post Routine. A single pre-routine measurement (La₀, VO₂) was also taken 1 minute before the start. The lower part of the figure outlines the sequence and timing of five separate trials: (1) full duet routine, (2) pre apnea 1 phase, (3) post apnea 1 phase, (4) pre apnea 2 phase, and (5) post apnea 2 phase, each separated by a 45-60 minute rest period. Rating of perceived exertion (RPE) was recorded during the post-routine phase after performing the full routine.

Each duet completed five protocol phases during a single training session, performing either the full routine or specific pre-defined segments (Fig 1). The five phases were performed in the following order: (1) the complete routine (full); (2) from the start of the routine to just before the first longest apnea (pre-apnea 1); (3) from the start to immediately after the first longest apnea (post-apnea 1); (4) from the start to just before the second longest apnea (pre-apnea 2); and (5) from the start to immediately after the second longest apnea (post-apnea 2). To minimize carryover effects and ensure adequate recovery, each of the five protocol phases was separated by a 45–60-minute rest interval (Fig 1), during which swimmers remained at the pool area, stayed warm, refrained from group training, and engaged in light active rest (gentle mobility and hand-only dryland marking) to prevent cooling.

The two longest apnea periods were identified by the swimmers’ coaches based on the specific choreography of each duet, and therefore varied between pairs.

At the start of the session, a coach-led specific 10-minute warm-up was performed. Immediately after, a 5-minute interval was used for equipment fitting and passive recovery at poolside, followed by measurement in the water of the pre routine rest during the minute immediately prior to the start of the full routine. For subsequent phases, approximately 15 minutes before each segment, swimmers completed their usual individual 10-minute warm-up, similar to the routines they perform before training or competition. The remaining 5 minutes were used for equipment refitting, and pre-phase rest was then recorded in the water during the minute immediately before each segment. To preserve ecological validity, neither the content of the coach-led warm-up nor the athletes’ subsequent warm-ups was standardized or controlled by the research team.

#### Cardiopulmonary assessment.

Oxygen uptake (VO₂) was measured breath-by-breath using a Hans-Rudolph 7400 oronasal mask (Hans Rudolph Inc., Shawnee, Kansas, USA) connected to a portable metabolic analyzer (K4b^2^, CosMed, Rome, Italy). VO₂ was measured directly during the minute preceding each routine phase. Immediately after the end of each phase, gas exchange was recorded continuously for the first three minutes of passive recovery, with participants remaining upright in the water, submerged up to the mid-sternum. These post-exercise values were used to retro-extrapolate and estimate the VO₂ peak corresponding to each segment.

Both swimmers in each duet were analyzed simultaneously using two independent K4b^2^ units. Before each phase, researchers, athletes, and technical staff jointly agreed on the exact location in the pool where each of the five routine phases would conclude. This endpoint was intentionally placed near the pool wall to allow researchers to be positioned in advance with the metabolic masks. Upon completion of each segment, swimmers were instructed to hold their breath until reaching the agreed-upon location, where the masks were immediately applied. Participants were instructed to take their first post-exercise breath directly into the mask to ensure data accuracy.

Exercise-phase VO₂ was estimated by linear regression to time zero using the first 20 seconds of the post-exercise VO₂ recovery curve [25], excluding the initial 5 seconds to reduce the influence of respiratory fluctuations and potential delays in oronasal mask application [26,27].

The mask was fitted quickly and securely to prevent air leakage and minimize delay in data acquisition. Prior to testing, swimmers and assistants were trained in the correct mask placement technique. The gas analyzers were calibrated before each test using certified reference gases (16% O₂ and 5% CO₂), and delay and volume turbine settings were adjusted using a 3-liter syringe in accordance with the manufacturer’s guidelines. Heart rate (HR) was continuously recorded using waterproof monitors (CardioSwim, Freelap, Switzerland) capable of capturing R–R intervals. To minimize instrumentation-related bias, participants used the devices during training sessions for several weeks before data collection. Pre-exercise heart rate (HRpre) was defined as the average HR recorded during the minute preceding the start of the routine, following the warm-up. For each routine segment, the following HR variables were recorded: maximum (HRpeak), minimum (HRmin), mean (HRmean), and range (HRrange), as well as post-exercise HR measured during the minute immediately following each segment.

#### Blood lactate concentration assessment.

Capillary blood samples (10 µL) were collected from the earlobe following the warm-up (5 minutes prior to the routine) and at 3 and 5 minutes post-exercise for each routine phase, except for the full routine, where additional samples were taken at 7 minutes post-exercise (Fig 1). The first post-exercise sample (at 3 minutes) was obtained immediately after completing VO₂ measurement. Lactate concentration was analyzed using a lactate photometer (Diaglobal DP100, Berlin, Germany), previously calibrated with lactate standards (2–20 mmol·L ⁻ ¹). The highest lactate value recorded after exercise was considered the post-exercise lactate peak (Lapeak).

#### Perceived exertion.

Rating of perceived exertion (RPE) was assessed using the Borg CR-10 scale. Participants were shown the scale visually and asked to rate their overall exertion between 3 and 5 minutes after completing the full routine. To minimize learning effects, RPE had been assessed during previous training and competition sessions prior to the evaluation day [28].

#### Video recording.

Video recordings of the duet routines were used to identify the exact start and end points of the full routine, as well as the beginning and end of each pre- and post-apnea phase. Recordings were made using a Sony HDR-XR550VE digital camera. Time verification and data synchronization were performed using LINCE PLUS software [29,30], based on predefined observational criteria for temporal alignment.

### Statistical analysis

Breath-by-breath VO₂ data and HR data, recorded continuously via R-R intervals, were interpolated to 1-second intervals using linear interpolation. VO₂ kinetics between phases were analyzed using a mono-exponential fitting model, widely recognized for its physiological validity and established application in VO₂ kinetics analysis [31,32]:

VO₂(t) = A + B (1 − e^ − t/τ) where VO₂(t) is oxygen uptake at time t; A is the VO₂ at the onset of exercise; B represents the amplitude of the increase in VO₂ during exercise (VO₂ peak − A), and τ is the time constant.

Descriptive statistics are presented as means and standard deviations (±SD). The Shapiro–Wilk test was used to assess the normality of the variables. Since the assumption of normality was not met, the Friedman test was applied to compare physiological responses across exercise phases. When significant differences were detected, post hoc pairwise comparisons were conducted using the Wilcoxon signed-rank test with Bonferroni correction. Effect sizes for these comparisons were calculated using Rosenthal’s r (r = Z/√N), where Z is the standardized test statistic and N the number of observations. Following conventional guidelines for correlation-type effect sizes [33], we interpreted r as small about 0.10, medium about 0.30, and large 0.50 or higher. These inferential analyses addressed the two a priori hypotheses stated in the Introduction (H1 and H2).

Post hoc power was estimated using repeated-measures ANOVA in G*Power 3.1 [34], as the software does not support non-parametric tests like the Friedman test. Given the similarity in design, repeated-measures ANOVA provides a valid approximation for within-subject analyses. Effect sizes were estimated from η² and used as input [34].

## Results

**Fig 2:**
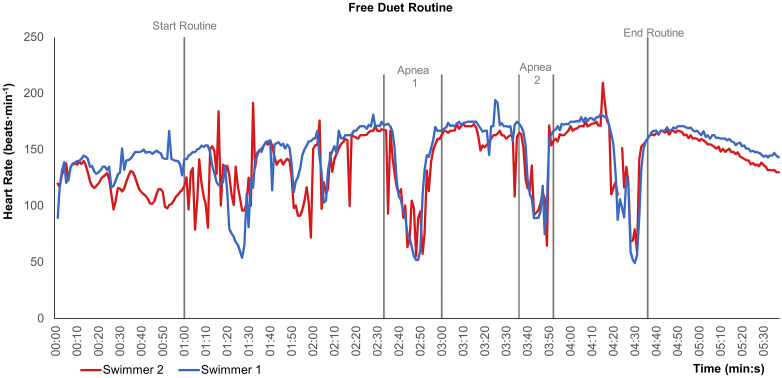
Time-synchronized heart rate recordings of two elite artistic swimmers before, during, and after a complete free duet routine, highlighting fluctuations during the two longest apnea phases. *Note.* Heart rate (HR) values (beats·min ⁻ ¹) recorded for two artistic swimmers during the routine. Vertical grey lines indicate key phases: start of routine, apnea 1, apnea 2, and end of routine.

The participants (n = 16) presented the following baseline mean ± standard deviation values prior to routine execution: VO₂ of 10.35 ± 3.43 mL·min ⁻ ¹·kg ⁻ ¹, HR of 110.78 ± 20.4 beats·min ⁻ ¹, and blood lactate concentration of 1.04 ± 0.49 mmol·L ⁻ ¹. During the full routine, HRpeak reached 203.83 ± 4.96 beats·min ⁻ ¹, HRmin was 71.85 ± 16.61 beats·min ⁻ ¹, and HRrange (peak – minimum) was 131.98 ± 15.21 beats·min ⁻ ¹. The rating of perceived exertion (RPE), recorded between 3 and 5 minutes post-routine, averaged 6.6 ± 1.5 (range: 4–9) (Fig 2).

HRmean and blood lactate increased across phases and showed significant differences, whereas VO₂ peak did not differ significantly between phases (Table 2). A similar trend was observed in the duration of each successive routine segment, as shown in Table 2.

**Table 2:** Physiological and temporal data from 16 artistic swimmers across different phases of the free duet routine.

Free Duet	Duration			VO_2_ peak			Heart Rate				Lactate			
(n = 16)	(s)			(mL·min^-1^·kg^-1^)			(beats·min^-1^)				(mmol·L^-1^)			
Full routine	197.7	±	10.4	61.8	±	15.1	156.1	±	10.8	#¶	5.9	±	1.4	#¶
Pre-apnea 1	66.7	±	20.6	54.7	±	6.5	139.7	±	16.6	$*&	3.7	±	1.6	&
Post-apnea 1	80.5	±	21.5	55.5	±	9.6	141.9	±	10.5	*&	3.8	±	2.2	&
Pre-apnea 2	170.6	±	24.7	60.2	±	11.1	153.6	±	10.3	#	4.9	±	1.9	
Post-apnea 2	183.5	±	23.0	60.7	±	10.8	157.4	±	14.6	#¶	5.7	±	2.0	

Note. Values are presented as mean ± standard deviation (SD). & = significantly different from full routine; # = significantly different from pre-apnea 1; ¶ = significantly different from post-apnea 1; $ = significantly different from pre-apnea 2; * = significantly different from post-apnea 2 (p < 0.05). For significant HR and lactate comparisons, effect sizes (Rosenthal’s r) were large according to Cohen’s benchmarks [33].

The HRrange increased progressively across the segmented phases: 112.71 ± 21.8 beats·min ⁻ ¹ in pre-apnea, 119.69 ± 27.0 beats·min ⁻ ¹ in post-apnea 1, 122.90 ± 18.9 beats·min ⁻ ¹ in pre-apnea 2, and 125.88 ± 20.0 beats·min ⁻ ¹ in post-apnea 2, reaching 131.98 ± 15.2 beats·min ⁻ ¹ in the full routine.

HRpeak also varied: 194.08 ± 13.0 beats·min ⁻ ¹ in the pre-apnea 1, 190.67 ± 15.8 beats·min ⁻ ¹ in post-apnea 1, 199.36 ± 9.5 beats·min ⁻ ¹ in pre-apnea 2, and 202.20 ± 11.0 beats·min ⁻ ¹ in post-apnea 2, with the highest value in the full routine (203.83 ± 5.0 beats·min ⁻ ¹). HRmin fluctuated between phases, ranging from 70.98 ± 21.2 to 81.37 ± 19.9, and was 71.85 ± 16.6 during the full routine.

**Fig 3:**
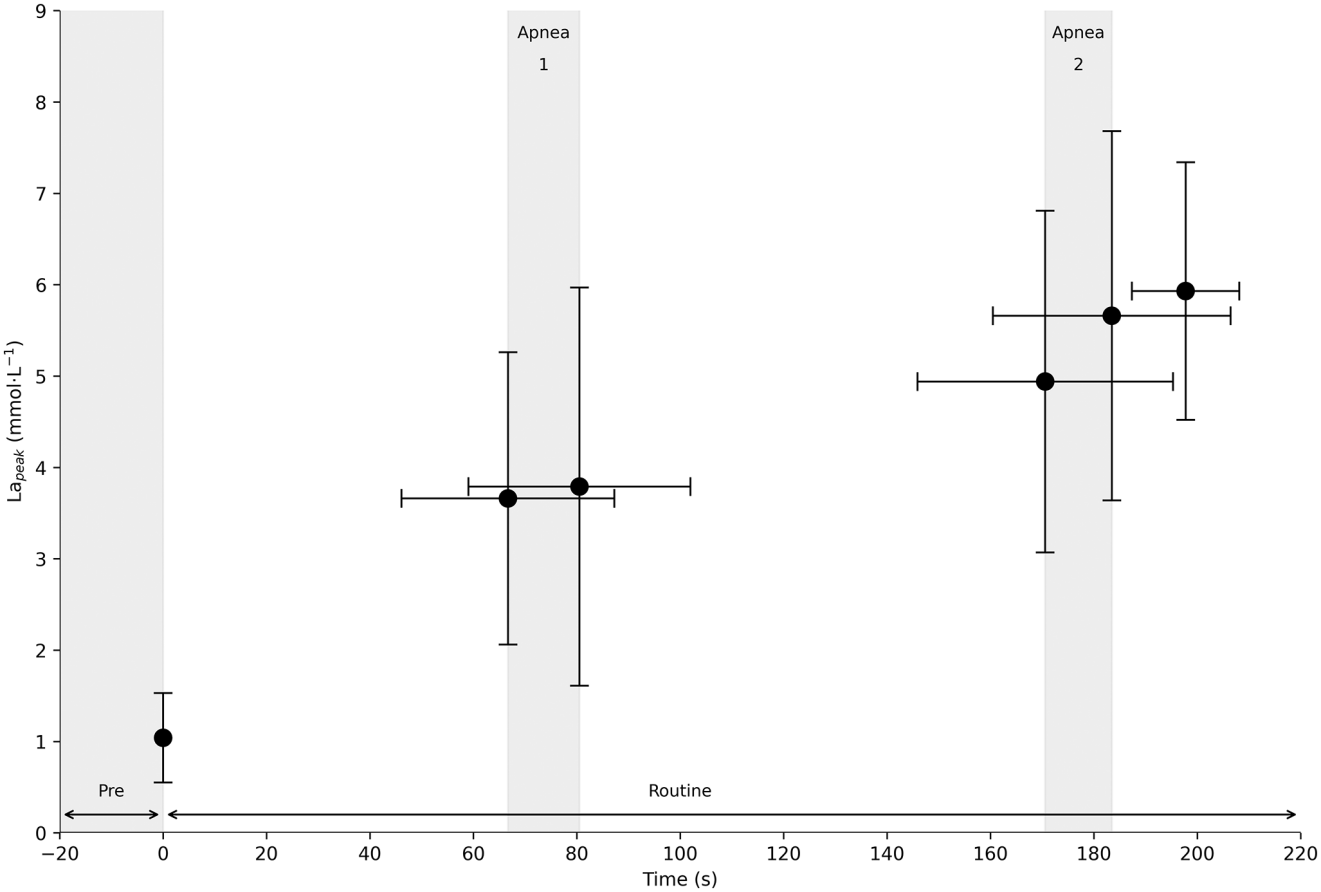
Lactate responses during full and segmented phases of a free duet routine in elite artistic swimmers. *Note.* Blood lactate concentration (mmol·L ⁻ ¹) measured after each phase of the free duet routine. Black circles represent mean values. Vertical and horizontal error bars indicate standard deviation for lactate concentration and sampling time, respectively. Shaded vertical regions correspond to apnea 1 and apnea 2.

Fig 3 shows the progression of peak blood lactate concentration across the different routine phases, illustrating a steady increase following each segment of the protocol.

**Fig 4:**
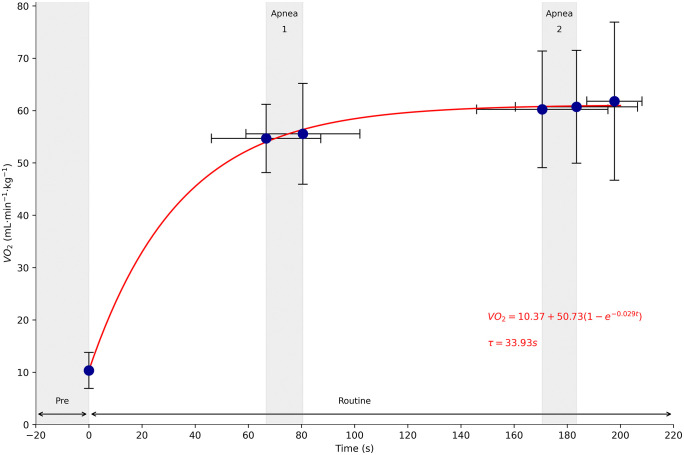
Oxygen uptake (VO₂) and VO₂ kinetics during full and segmented phases of a free duet routine in elite artistic swimmers. *Note.* VO₂ (mL·min ⁻ ¹·kg ⁻ ¹) measured after each phase of the free duet routine. Blue circles represent mean values. Vertical and horizontal error bars indicate standard deviation for VO₂ and sampling time, respectively. The red curve represents a mono-exponential model fitted to the data, with the time constant (τ) indicated. Shaded vertical regions correspond to apnea 1 and apnea 2.

Fig 4 displays VO₂ across phases. Pre- and post-apnea values within each segment were similar and differences were not statistically significant. A mono-exponential curve was fitted to the data to model VO₂ kinetics during exercise. In this model, the time constant (τ) represents the time required to reach 63% of the final VO₂ value; in the present study, τ was calculated as 33.9 seconds. The figure also highlights the similarity between pre- and post-apnea measurements observed within each segment.

## Discussion

Continuous measurement of oxygen uptake (VO₂), blood lactate, and heart rate (HR) during artistic swimming routines is limited by the frequent immersion of swimmers. The segmentation strategy employed in this study enabled the reconstruction of a temporal physiological profile across a full competitive free duet routine in elite artistic swimmers. Across the routine, physiological responses increased. Heart rate and blood lactate showed statistically significant phase-related increases, whereas VO₂ did not differ significantly between phases. Pre versus post apnea comparisons were also non-significant for HR, VO₂, and blood lactate. Under the conditions of this study, we did not observe a distinct physiological effect attributable to the apneas.

Previous studies have reported VO₂ peak values in artistic swimmers ranging from 43 to 52.4 mL·min ⁻ ¹·kg ⁻ ¹, using both specific and non-specific protocols [8,17–19,21–23]. In contrast, the VO₂ peak recorded in our study (61.8 ± 15.1 mL·min ⁻ ¹·kg ⁻ ¹) represents the highest reported to date in this discipline. This likely reflects a combination of factors: all participants were elite athletes, some of them World Championship medalists, with consistently high training volumes (approximately 30 hours per week), which may have contributed to enhanced aerobic capacity. Such elevated values are consistent with studies linking VO₂ peak to performance [8,17–19,21,23].

VO₂ rose rapidly during the routine, reaching 90% of VO₂ peak within approximately 67 seconds. The calculated time constant (τ = 33.9 s) aligns with values reported for healthy but non-specialized populations [32] and is comparable to those measured during maximal 400-m front crawl test (30.6 ± 4.9 s) [31]. In contrast, elite endurance athletes show τ values as low as 8–10 seconds, placing our results in perspective relative to physiological upper limits. A mono-exponential model was used due to its physiological validity and established application in VO₂ kinetics analysis [32].

Artistic swimming is characterized by high metabolic demands. In the present study, the mean post-routine blood lactate concentration was 5.93 ± 1.41 mmol·L ⁻ ¹, closely matching the value reported by Bante et al. [21] (5.7 mmol·L ⁻ ¹) following a simulated routine approximately 20 seconds shorter than the free duet routines performed here (177 ± 7 s). However, most previous studies have reported higher post-exercise lactate levels, typically ranging from 7.5 to 11.5 mmol·L ⁻ ¹ [3,8,19,22,23].

One possible explanation for the lower blood lactate concentrations observed in our study is that three of the routines analyzed were not fully completed at the time of testing, with durations shortened by 4–14 seconds. This decision was made to preserve the ecological validity of high-performance training and to minimize interference with athletes’ competition preparation, particularly for duets from the junior Spanish national team who were still refining their choreography. As a result, the average total duration of the routines was 197.7 ± 10.4 seconds. Given the progressive accumulation of blood lactate throughout a routine, it is plausible that those additional seconds would have led to values more in line with previous reports.

Another contributing factor may be the notably higher VO₂ peak values observed in our sample. A higher VO₂ peak is associated with a greater reliance on aerobic metabolism during exercise [35]. Therefore, under similar energetic demands, swimmers with superior aerobic capacity would be expected to engage less glycolytic activity, resulting in lower post-exercise blood lactate levels.

Figura et al. [36] reported lower post-exercise blood lactate values than those observed in our study, with an increase of 3.37 ± 0.5 mmol·L ⁻ ¹ from a rest of 1.46 ± 0.4 mmol·L ⁻ ¹, resulting in a final concentration of approximately 4.8 mmol·L ⁻ ¹. This discrepancy may be partially explained by the evolution of artistic swimming over the past two decades, during which routines have become markedly more demanding, incorporating increasingly complex lifts, jumps, and acrobatic elements [1]. Notably, our study was conducted 18 years after that of Figura et al. [36], reflecting substantial changes in technical requirements and overall performance intensity.

HR response during artistic swimming routines has also been explored in previous research. In agreement with Rodríguez-Zamora et al. [3], our findings confirm the presence of wide range of HR values, with peak values alternating with bradycardic episodes that coincide with the execution of high-intensity figures under apnea.

Pre-exercise heart rate (HRpre) in our free duet routines was lower than the values reported in previous studies [3,37]. An anticipatory HR response mediated by sympathetic activation has been well documented and is known to occur similarly in training and competition settings, with greater intensity in junior compared to senior swimmers [3,37].

In this study, pre-routine measurements were taken following a warm-up guided by the technical staff but not standardized by the research team, which may have contributed to additional physiological activation, combined with anticipatory effects described in the literature, these factors may account for the relatively elevated pre-exercise VO₂ and heart rate values observed. Miyamoto et al. [38] demonstrated increases in both parameters during the pre-exercise phase, attributed to feedforward mechanisms from the central nervous system. Complementarily, Elstad et al. [39] confirmed that heart rate rises immediately before exercise onset, reflecting a cardiovascular anticipatory response. In addition, repeated exposure to voluntary apneas, as occurs in artistic swimming, may modulate anticipatory ventilatory responses, as suggested by Wood et al. [40]. This pattern has also been documented in artistic swimmers during competitive settings [3].

Furthermore, previous studies have reported resting heart rates exceeding 130 bpm in similarly trained swimmers prior to both training and competition routines, with no significant differences between contexts [3].

The direct effect of apneas on blood lactate concentration has been specifically studied only by Figura et al. [36], who observed increases of 1.15 ± 0.27 mmol·L ⁻ ¹ and 1.32 ± 0.12 mmol·L ⁻ ¹ following the Heron and Eiffel Walk figures, each lasting approximately 45 ± 1.5 seconds. In contrast, our study did not reveal significant differences between pre- and post-apnea lactate values. After the first main apnea (mean duration: 13.8 seconds), the increase in blood lactate was only 0.13 mmol·L ⁻ ¹. Following the second apnea (12.9 seconds), the observed increase was 0.72 mmol·L ⁻ ¹.

Butler and Jones [41] proposed the concept of the “diving lactate threshold” (DLT) to define the apnea duration beyond which blood lactate concentration begins to rise.

During apnea, as oxygen reserves are depleted, the body shifts progressively toward glycolytic metabolism, eventually reaching a point where lactate production exceeds its clearance capacity [42]. The rate of VO₂ decline during apnea is influenced by multiple factors, with exercise intensity being among the most significant [43–45]. In artistic swimming, the execution of figures during apnea phases maintains a high metabolic demand. Therefore, the absence of a marked increase in blood lactate concentration in our study may be attributed to the relatively short duration of the apneas, which likely did not deplete oxygen stores sufficiently to trigger a greater reliance on anaerobic glycolysis.

In artistic swimming, the diving response has been shown to attenuate the exercise-induced increase in heart rate during apnea [3]. Apnea also leads to a reduction in stroke volume, further decreasing cardiac output during these phases [46,47]. As a result, central components of oxygen delivery are diminished, suggesting that VO₂ during apnea is primarily sustained by peripheral mechanisms. Our findings support this interpretation, as VO₂ measured at the end of apnea did not differ significantly from pre-apnea values. This aligns with the observations of Grossman et al. [48], who reported that sustained VO₂ during apnea is maintained through increased muscle deoxygenation, constrained by the diving response. Additionally, the rapid cardiovascular adjustments that occur immediately after apnea [49] likely facilitate the restoration of VO₂ during subsequent eupneic periods.

This study is not without limitations, the most notable of which include:

(1) Continuous measurement of VO₂ during apnea phases was not possible due to frequent submersions inherent to artistic swimming. Consequently, VO₂ during apnea was inferred from values recorded immediately before and after each apnea, supplemented by previous evidence on the physiological responses to breath-hold activity. (2) The apneas analyzed were relatively short (<20 seconds), which may explain the absence of significant changes in blood lactate concentration. As prior studies suggest, longer apnea durations may be required to reach the diving lactate threshold (DLT), limiting the generalizability of our findings to routines involving more prolonged breath-holds. (3) Some routines were not completed in full at the time of data collection, in order to preserve the ecological validity of high-performance training and avoid interfering with athletes’ competition preparation. (4) Although 20 swimmers were initially recruited, two duets (n = 4) were excluded due to substantial signal loss in HR measurements caused by underwater movement artifacts, which rendered their data unsuitable for analysis. (5) Rest immediately before each execution was measured in water rather than at a quiet seated baseline. Rest intervals allowed light mobility and hand only dryland marking, and warm ups were coach led or athlete led and not standardized by the research team. These factors may have increased anticipatory heart rate and oxygen uptake.

Future research should focus on the effect of longer apneas on lactate production and their impact on subsequent VO₂ levels. In addition, near-infrared spectroscopy (NIRS) could enable continuous monitoring of muscle (SmO₂) and cerebral (ScO₂) oxygen saturation during routines, building on the work of Jones and Cooper [9]. Applied specifically to artistic swimming, NIRS may enhance our understanding of the peripheral factors regulating VO₂ during apnea. Furthermore, the diving lactate threshold (DLT) could be examined in relation to the intensity and duration of individual figures. Assessing DLT across different elements and performance levels may offer valuable insights for optimizing training prescription.

## Conclusions

This study demonstrates that artistic swimming routines elicit a rapid physiological response, with 90% of VO₂ peak reached within approximately 67 seconds. Significant increases in blood lactate concentration and heart rate were observed throughout the routine, particularly in the later phases (e.g., blood lactate: from 3.66 mmol·L ⁻ ¹ in pre-apnea 1 to 5.93 mmol·L ⁻ ¹ after the full routine, p < 0.05; HRmean: from 139.68 to 141.89 beats·min ⁻ ¹, p < 0.05). No significant differences in VO₂ were detected between phases, and pre- versus post-apnea comparisons were also non-significant, suggesting that oxygen uptake remains relatively stable across the routine.

Blood lactate concentration showed similarly limited variation (ΔLa: 0.13 and 0.72 mmol·L ⁻ ¹), likely due to the brief duration of the apneas, which may have been insufficient to induce a metabolic shift toward glycolysis.

Peak VO₂ (61.79 ± 15.1 mL·min ⁻ ¹·kg ⁻ ¹) and end-of-routine blood lactate (5.93 ± 1.41 mmol·L ⁻ ¹) reflect both substantial aerobic and anaerobic contributions.

These findings suggest that the physiological demands of elite-level free duet routines may be primarily influenced by sustained exercise intensity and the cumulative effect of repeated apneas, rather than by isolated breath-hold events.
